# The Psychophysiological Regulation of Pacing Behaviour and Performance Fatigability During Long-Distance Running with Locomotor Muscle Fatigue and Exercise-Induced Muscle Damage in Highly Trained Runners

**DOI:** 10.1186/s40798-018-0143-2

**Published:** 2018-07-10

**Authors:** Andreas Venhorst, Dominic P. Micklewright, Timothy D. Noakes

**Affiliations:** 1 Department of Human Biology, Division of Exercise Science and Sports Medicine, University of Cape Town, Newlands, 7725 South Africa; 2 School of Sport, Rehabilitation and Exercise Sciences, University of Essex, Colchester, CO4 3SQ UK

**Keywords:** Locomotor muscle fatigue, Exercise-induced muscle damage, Pacing behaviour, Endurance performance, Long-distance running, Central regulation, Psychophysiology, Perceived fatigability, Performance fatigability

## Abstract

**Background:**

Locomotor muscle fatigue (LMMF) and exercise-induced muscle damage (EIMD) are common conditions experienced during long-distance running due to the pooled effect of mechanical and metabolic strain on the locomotor muscles. However, little is known about the instant effects of combined LMMF and EIMD on pacing behaviour and performance during the decisive final stages of ‘real-world’ long-distance running events.

**Methods:**

Twenty-two highly trained runners (11 females) completed two maximal self-paced 20-km treadmill time trials in a counterbalanced crossover design: (A) in a tapered condition and (B) with LMMF and EIMD. Indicators of muscle damage, muscle metabolic strain, and endocrinological stress were assessed to investigate the physiological effects, and a three-dimensional framework of perceived fatigability was applied to investigate the perceptual effects of running with LMMF and EIMD on performance fatigability.

**Results:**

LMMF and EIMD caused restrictions in work capacity and medium increases in blood leucocyte and neutrophil count, interleukin-6, and cortisol concentrations, collectively constituting a physiological milieu likely not conducive to high performance. LMMF and EIMD further caused large increases in perceived physical strain and large decreases in valence as well as large increases and decreases in action crisis and flow state, respectively.

**Conclusions:**

Under the constraint of amplified physical duress, findings are suggestive of heuristic and rational antecedents in the goal disengagement process. Dynamic changes in physiological and perceptual effects of LMMF and EIMD are hypothesised to underpin the observed alterations in pacing behaviour and performance fatigability during long-distance running. The applied three-dimensional framework provides a more comprehensive understanding of strain-perception-thinking-action coupling in centrally regulated and goal-directed exercise behaviour.

**Electronic supplementary material:**

The online version of this article (10.1186/s40798-018-0143-2) contains supplementary material, which is available to authorized users.

## Keypoints

- Little is known about the instant effects of combined locomotor muscle fatigue (LMMF) and exercise-induced muscle damage (EIMD) on pacing behaviour and performance fatigability during the decisive final stages of ‘real-world’ long-distance running events.
- Running with LMMF and EIMD caused medium increases in cardiovascular, respiratory, and metabolic responses as well as a non-adaptive physiological distress response, collectively constituting a physiological milieu not conducive to high performance.
- Running with LMMF and EIMD further caused large increases in perceived physical strain and decreases in valence as well as deteriorations in action crisis and flow state suggestive of heuristic and rational antecedents in the goal disengagement process.
- Dynamic changes in physiological and perceptual effects are hypothesised to underpin the observed alterations in pacing behaviour and performance during long-distance running with LMMF and EIMD.

## Background

Unaccustomed muscular exertion during training for, and competition in, prolonged endurance events can result in significant locomotor muscle fatigue (LMMF) and exercise-induced muscle damage (EIMD) [1]. Particularly, activities involving muscle-lengthening contractions such as running are more likely to induce muscle damage due to high muscular strain coinciding with low neuromuscular recruitment. Thus, the underlying cause of EIMD is suggested to be largely mechanical in nature [2]. However, EIMD also occurs in activities predominantly involving muscle shortening contractions such as swimming and cycling. Metabolic deficiencies are therefore also suggested to play a significant role in muscle damage when metabolic strain outweighs mechanical strain [3]. Accordingly, EIMD is a commonly experienced condition in long-distance running events characterised by a pooled effect of mechanical and metabolic strain on skeletal muscle injury [1, 4–6].

So far, research has focussed on the physiological consequences of EIMD day(s) after inducing it and therefore largely on delayed onset of muscular soreness (DOMS). For example, review articles discussed indicators of muscle damage, the repeated bout effect, and sex differences [7]; impact on neuromuscular function and excitation-contraction coupling, sarcomere insertion, and damage to contractile machinery and selective fibre types [8]; impaired metabolism [3]; and inflammatory and cytokine response [9, 10], as well as molecular and cellular mechanisms of damage and recovery [11].

More specifically, EIMD can negatively impact endurance performance by altering cardiorespiratory, metabolic, biomechanical, and thermoregulatory variables. This includes but is not limited to tachycardia [12, 13], augmented ventilatory response and oxygen consumption [14, 15], attenuated peak oxygen uptake and ventilatory threshold [16], increased intramuscular carbohydrate oxidation [17], increased blood lactate concentrations [18–20], compensatory modifications in running kinematics [21], decreased running economy [18, 22], and increased core temperature [23]. Critically, differential responses in these variables are more likely to occur once ventilatory threshold intensities are surpassed. In addition, the effect sizes of individual variables are only small to moderate, which might explain why experimental studies with usually small sample sizes of ≤ 10 participants do not consistently reach statistical significance, thereby erroneously suggesting conflicting findings or null effects.

Furthermore, these debilitating effects of increased physiological strain on endurance performance are accompanied by debilitating effects of increased perceived fatigability (defined as the changes in perceptions that regulate the integrity of the performer [24]). This includes and is currently limited to increased perception of effort and perceived exertion [12, 13, 15, 25] and exercise-induced pain [26, 27]—two of several determinants in the central regulation of pacing behaviour and endurance performance [28].

However, most of the hitherto mentioned studies investigated the impact of EIMD day(s) *after* inducing it and are therefore confounded by the pronounced systemic inflammatory response characterising DOMS. Accordingly, little is known about the instant effects of combined LMMF and EIMD on pacing behaviour and endurance performance as experienced *during* the decisive final stages of ‘real-world’ long-distance running events.

In contrast, two studies investigated the instant effects of LMMF and mild EIMD (average force loss of 19%) on high-intensity cycling exercise of ≈ 15 min in moderately trained participants. Marcora et al. [13] used a time-to-exhaustion protocol at 80% of peak power output and observed significant decreases in endurance performance as well as significant increases in tachycardia, tachypnoea, and perception of effort during the fatigue trial. de Morree and Marcora [29] used a 15-min time trial and observed large decreases in average power output (without differences in pacing behaviour), large decreases in vastus lateralis electromyogram activity over time, large reductions in blood lactate concentrations at the end, and large increases in perception of effort at the start of the intervention time trial. Both authors proposed a mediatory role of perception of effort in the relationship between LMMF and mild EIMD and endurance performance consistent with increased central motor command and reafferent signalling.^1^ However, the findings are of limited use in understanding the psychophysiological regulation of pacing behaviour and performance in high-performance long-distance runners, as they used cyclists rather than runners, moderately rather than highly trained athletes, and short rather than prolonged time trials, and further solely assessed effort perception rather than using a more comprehensive multi-dimensional approach.

Recently, a three-dimensional framework of centrally regulated and goal-directed exercise behaviour was proposed, which emphasises the dynamic and complex interplay of sensory, affective, and cognitive processes that underpin perceived fatigability [30]. This framework more completely accounted for perception-thinking-action coupling in response to psychological distress (i.e. falling behind a performance matched competitor) than the traditional Gestalt concept of perceived exertion [31]. Here, the proposed framework was applied under the constraint of physical distress investigating the psychophysiological determinants of pacing behaviour and endurance performance in highly trained long-distance runners during two self-paced maximal 20-km treadmill time trials: (A) running in a tapered condition versus (B) running with LMMF and EIMD produced by a standardised drop-jump protocol. We hypothesised that the physiological and perceptual effects of amplified physical strain are associated with dynamic changes in performance fatigability (defined as the observed decline in an objective measure of endurance performance over a discrete period of time [24]) and that strain-perception-thinking-action coupling of sensory, affective, and cognitive processes more completely accounts for the dynamic changes in observed pacing behaviour.

## Methods

### Participants

Twenty-two runners, 11 females, estimated to fit performance level 4 criteria [32], were recruited from local running clubs via email distribution of digital flyers. Main inclusion criteria were a half marathon personal best of less than 1 h:40 min and 1 h:25 min for female and male runners, respectively. The 15% absolute performance difference accurately reflects the average sex difference in the TOP-100 finishing times of an international half marathon race event (www.twooceansmarathon.org.za) hosted locally. Performance level categorisation of each participant was subsequently verified according to criteria by DePauw and colleagues [32, 33]. Sample size estimation using conventional methods (one-tailed alpha of < .05, *β* > .80, and large effect size) and G*Power 3 software (G*Power, Version 3.1.9.2, Kiel, Germany) indicated overall 20 participants would be needed. All participants provided prior written informed consent to the procedures used in this study, which were approved by the institutional ethics committee and carried out in accordance with the Declaration of Helsinki**.**

### Study Design

Participants completed in total four visits including three maximal self-paced 20-km treadmill time trials over a simulated profiled course. A 10-km time trial took place after preliminary testing and acted as a submaximal familiarisation time trial (FTT). Before returning for their second visit, participants were instructed to log their training and diet for 48 h prior to the baseline time trial (BTT) and to prepare in a way that resembled their routine before an important race. They were then advised to repeat this ‘mini taper’ for the following experimental time trials. Participants then completed the intervention time trial (ITT) and control time trial (CTT) in a counterbalanced AB/BA crossover design. The ITT was preceded by a standardised drop-jump protocol (for details, see the “Drop-Jump Protocol” section below), while the CTT was preceded by a rest period of equal length. A washout period of 4 weeks took place before the crossover trial to (A) allow for complete recovery of muscle damage and (B) control for menstrual cycle in female runners (two were using hormonal contraception and two were amenorrhoeic), who were instructed to schedule experimental trials 5 to 9 days after the start of menses (early follicular phase).

### Procedures

#### Preliminary Measurements

During the first laboratory attendance, each participant had their age, stature, and body mass recorded. Body fat percentage was estimated using the seven skinfold method [34].

#### Peak Treadmill Running Speed Test

A peak treadmill running speed (PTRS) test with peak oxygen consumption (VO_2peak_) was performed on a motor-driven treadmill (Viasys LE500 CE, Hoechberg-Wuerzburg, Germany). The treadmill gradient was set to 1% simulating the energetic costs of outdoor running [35]. After a 10-min self-paced warm-up, participants started the PTRS at 10 and 11 km h^−1^ for female and male runners, respectively, after which speed increased stepwise by 0.5 km h^−1^ every minute. PTRS was calculated as the last completed stage added to the product of the speed increment and the completed fraction of the incomplete stage. During the progressive exercise test, expired ventilation volume (*V*_*E*_), oxygen uptake (VO_2_), and carbon dioxide production (VCO_2_) were measured with an online breath-by-breath gas analyser and pneumotach (Cosmed Quark b^2^, Rome, Italy). Calibration took place before each trial according to the manufacturer’s instructions using a 3-l syringe (Hans Rudolph 5530, Inc., Kansas City, MO, USA) and a gas mixture of known composition (5.05% CO_2_; 15.97% N_2_). Peak oxygen uptake was determined as the highest recorded VO_2_ measurement averaged over 30 s. The first ventilatory threshold and respiratory compensation point were determined according to the methods described by Lucia et al. [36]

#### Maximum Isokinetic Power Output Test

Maximum isokinetic power output of the quadriceps and hamstring muscles of the dominant leg was measured using the Biodex 3 Isokinetic dynamometer (Biodex Medical Systems, Shirley, NY, USA). Participants were familiarised with the procedure before the FTT and BTT. Participants were seated with their arms crossed over their chest, the hip flexed at 90°, and a knee angle of 90° from full leg extension. The lateral condyle of the femur was aligned with the rotational axis of the dynamometer, and the ankle was secured to the lever arm of the dynamometer using a padded Velcro strap. Shoulder and waist straps were used to fixate joint positions during trials. Standardisation was enhanced by performing gravity correction. After a warm-up set of increasing intensity (50, 70, and 90%), participants performed six maximal isokinetic contractions at 120° s^−1^. Isokinetic measurements were chosen due to the repetitive cyclic nature of running specific muscle contractions, and the above movement velocity was selected as it more closely resembles running specific contraction speeds as well as provides a trade-off between peak and explosive power output usually measured at < 60 s^−1^ and > 180° s^−1^, respectively. Power output curves were displayed on a screen in front of the participant, and verbal encouragement was given during maximal contractions.

During the experimental trials, maximum isokinetic power output was assessed twice: (1) after the 10-min self-paced warm-up and (2) within 2 min after completing the drop-jump protocol and control period, respectively.

#### Running Economy Test

Assessment of running economy took place before each of the three maximal 20-km time trials. After 5 min of running at a constant speed of 10 and 11 km h^−1^ for female and male runners, respectively, participants ran for 5 min at a speed corresponding to ∆ 1/3 between the first ventilatory threshold and the respiratory compensation point determined during the PTRS. The average values of breath-by-breath VO_2_ and VCO_2_ during the final minutes were used to calculate the oxygen cost and energy cost of running. Updated non-protein respiratory quotient equations were used to estimate substrate use (g min^−1^) during the monitored period [37]. The mean energy content of the metabolised substrates was then calculated according to the methods described by Jeukendrup and Wallis [38], scaled to body mass (BM^−1^) and expressed per kilometre (km^−1^) [39].

#### Time Trial Procedure

Participants performed three maximal self-paced 20-km time trials over a simulated profiled course on the same treadmill. A customised course profile was written using h/p/cosmos para-graphics® software (Version 2.6.14, h/p/cosmos sports and medical GmbH, Nussdorf-Traunstein, Germany) simulating a 20-km long time trial with two uphill sections of 2 km length and a gradient of 7%, starting after 4 and 12 km, respectively. The uphill sections were immediately followed by two downhill sections of the same length and gradient. Participants were assisted with gradient-dependent alterations in running speed, but custom-made modifications of the treadmill enabled participants to self-select running speed at any time with a handheld remote control in 0.1 km h^−1^ increments. Only course profile, gradient, and distance covered were displayed to participants during each time trial. Participants were instructed to complete the time trial in the shortest possible time, but no verbal encouragement was given during the time trial itself.

A fan was placed 1.5 m in front of the participants, and the fan level was adjusted in accordance with the course profile to simulate speed-related alterations in peripheral cooling. During the uphill sections, fans were set on level 1, during the flat sections on level 2, and during the downhill sections on level 3, respectively, creating average wind speeds of 3.15, 3.80, and 4.25 ms^−1^. Participants were permitted to consume water ad libitum, and a commercially available carbohydrate drink (Enduren™ Endurance Energy Drink) was provided at 2-km intervals and upon request for an average rate of ≈ 60 g h^−1^. All experimental trials were conducted under stable climatic conditions (temperature 20.5 ± 0.7 °C; humidity 58.3 ± 5.1%) and commenced at 8 am to control for diurnal variations.

During each running economy test and time trial, speed, gradient, and distance covered were recorded and stored at 1-s intervals using h/p/cosmos para-graphics® software. Heart rate was recorded at 2-s intervals throughout each trial by telemetry (Suunto® T6, Suunto Oy, Vantaa, Finland). Performance and heart rate data were subsequently analysed with TrainingPeaks™ analysis software (WKO edition+, Version 3.0, Lafayette, CO, USA). All continuously captured data were averaged into 2-km bins before statistical analyses were performed.

#### Drop-Jump Protocol

During the intervention trial only, participants performed muscle-lengthening contraction exercise consisting of 100 drop-jumps from a step of 45 cm height [40] known to induce force losses consistent with mild EIMD and without confounding effects of significant muscle metabolite accumulation (blood lactate concentration [mmol/l]: CTT = 1.5 ± 0.6 vs ITT = 1.4 ± 0.6) and cardiovascular demands (heart rate [bpm]: CTT = 65 ± 10 vs ITT = 100 ± 11) [29]. Participants dropped to an approximate knee angle of 90° before jumping upward as high as possible. Drop-jumps were performed every 20 s for a total time of ≈ 33 min. Vertical displacement of the centre of mass (COM) was calculated by means of a floor-embedded force plate (AMTI, Watertown, MA, USA) and a customised programme written in MATLAB® (R2013a, The Mathworks Inc., Natick, MA, USA) determining vertical take-off velocity of COM [41]:

#### Equation 1: determining jump-height from vertical take-off velocity

$$ \mathrm{Jump}-\mathrm{height}={\mathrm{TOV}}^2\kern0.5em 2{g}^{-1}, $$

where TOV = vertical velocity of COM at take-off, *g* = 9.81 ms^−2^.

Before and after the drop-jump protocol and rest period, perceived muscle discomfort and unpleasantness were assessed by means of 100 mm visual analogue scales (VAS) ranging from ‘no muscle discomfort/unpleasantness at all’ to ‘unbearable muscle discomfort/unpleasantness’. The extent to which participants experienced DOMS in the 84-h recovery period after experimental trials was assessed using a 7-point Likert-type scale [42].

### Measurements

#### Measures of Perceived Fatigability

During the final minutes of the running economy test and at 2-km intervals during each time trial, participants provided ratings on the following four single-item scales presented in random order. All scales were anchored during the PTRS test.

The 15-point (6–20) Borg scale with the indicator terms ‘light’ and ‘strong’ was used to approximate perceived physical strain by phrasing ‘How strong are the physical sensations from your legs, lungs, and body?’ Participants were instructed to include only the subjective perception of physical sensations *caused by* the task and to focus on location, quality, and intensity of physical sensations before returning an overall perceived physical strain score.

The 15-point (0–14) Borg scale with the indicator terms ‘easy’ and ‘hard’ was used to approximate perceived mental strain by phrasing ‘How difficult is it to run at this pace?’ Participants were instructed to include only perceived task difficulty and mental effort *invested into*/*required to continue with* the task before returning a perceived mental strain score.

In line with recent recommendations [43], a detailed three-tiered justification process has been outlined for measurement selection in the assessment of dynamic changes in core affective state during prolonged endurance exercise [44]. Ultimately, the 11-point (− 5 ‘very bad’ to 0 ‘neutral’ to + 5 ‘very good’) Feeling Scale (FS) and 6-point (1 ‘low activation’ to 6 ‘high activation’) Felt Arousal Scale (FAS) were chosen to approximate dynamic changes in valence and felt activation, respectively [45, 46].

The Action Crisis Scale (ACRISS) and short Flow State Scale (FSS) were administered during the 30-min recovery period retrospectively measuring the extent to which participants experienced a shift from an implemental to a deliberative mindset. Both scales were administered for seven sections of the profiled TT course (three flat, two uphill, and two downhill) and rated on 5-point Likert-type scales.

The ACRISS comprises six items: conflict, setbacks, implemental disorientation, rumination, disengagement impulses, and procrastination [47]. The internal consistency estimate of reliability was good with mean Cronbach’s alpha of 0.83 ± 0.08 and 0.89 ± 0.04 during the control and intervention time trial, respectively.

The FSS comprises nine items: challenge-skill balance, action-awareness merging, clear goals, unambiguous feedback, concentration on task at hand, sense of control, loss of self-consciousness, transformation of time, and autotelic experience [48]. The internal consistency estimate of reliability was good with mean Cronbach’s alpha of 0.81 ± 0.06 and 0.81 ± 0.07 during the control and intervention time trial, respectively.^2^

#### Physiological Measures

Venous blood samples from a superficial antecubital vein were taken at rest, after the drop-jump protocol, after the running economy test, halfway through and at the end of experimental time trials as well as after the 30-min recovery period. Blood samples were placed into four different pre-chilled Vacutainers, respectively containing (A) serum clot activator for the analysis of cortisol, (B) potassium oxalate and sodium fluoride for the analysis of lactate concentrations, and (C) K_2_-ethylenediaminetetraacetic acid (EDTA) for the analysis of interleukin-6, differentiated white blood cell count, haemoglobin, and haematocrit. Where appropriate, samples were inverted five times, immediately centrifuged at 3000 rpm at 4 °C for 10 min, plasma/serum pipetted off, and kept on ice until stored at − 80 °C for subsequent analysis. Plasma lactate concentrations were determined using glucose oxidase method (YSI 2300 STAT PLUS, Ohio, USA). Serum cortisol was determined using an automated chemiluminescence system (Architect iSR, Abbott Diagnostics, IL, USA) with conventional reagent kits and calibrators. Interleukin-6 was analysed by means of enzyme-linked immunosorbent assay (ELISA) (eBioscience, Bender MedSystems, Vienna, Austria). Differentiated white blood cell count, haematocrit, and haemoglobin were determined by Lancet Laboratories using conventional methods. The degree of haemoconcentration was calculated, and all blood samples were subsequently corrected for plasma volume changes [49].

#### Measures of Performance Fatigability

Besides the assessment of global time trial performance between experimental conditions, this study aimed to analyse the decline in objective measures of endurance performance over time. The second-half to first-half split time quotient was calculated as a crude assessment of the degree of positive or negative pacing, and repeated measures of 2-km split time intervals were used to locate onset and extent of performance fatigability. For a clearer graphical presentation, dynamics in perceived fatigability were further indicated by the percentage increase in split times during ITT compared to CTT and accordingly assessed via one-way repeated measures ANOVA.

### Statistical Analysis

Data were tested for assumptions, normality, equality of variances, equality of covariance matrices, and sphericity where appropriate. Independent samples *t* tests were used to analyse inter-individual differences in parameters between the sexes, and paired samples *t* tests were used for intra-individual comparisons in parameters between experimental trials. Between-group effect sizes were calculated using Cohen’s *d* (trivial < 0.20, small 0.21–0.60, medium 0.61–1.20, large 1.21–2.00, very large 2.01–4.00, and near perfect > 4.00). A non-parametric Mann-Whitney *U* test was performed when the assumption of equality of variance was violated. Confidence intervals and magnitude-based inferences for meaningful differences between time trial performances were derived from *p* values in accordance with Hopkins [50]. Two-way repeated measures ANOVAs were used to compare changes in parameters over time between experimental trials. Significant interaction effects were followed up with Bonferroni correction procedure. A Greenhouse-Geisser epsilon adjustment was made when sphericity was violated. Aligned rank transformation procedure (ARTool software 1.5.1, Washington, USA) was used to perform non-parametric factorial analysis on all perceptive data and when assumptions of parametric tests were violated [51]. All ANOVA effect sizes were calculated as partial eta squared (*η*_*p*_^2^) and classified as small 0.02–0.13, medium 0.13–0.26, and large > 0.26. All data are presented as mean ± one standard deviation, and an alpha level of < .05 (two-tailed) was used to indicate statistical significance.

## Results

### Participant Characteristics

Anthropometric characteristics and performance parameters of female (*n* = 11) and male (*n* = 11) participants are listed in Table 1.

**Table 1:** Sex comparison of anthropometric, training, and performance data

	Male (*n* = 11)	Female (*n* = 11)	*p* value	Effect size
Descriptive data				
Age (years)	29 ± 7	28 ± 10	n.s.	–
Stature (cm)	173 ± 7	163 ± 5	.001	Large
Body mass (kg)*	64.7 ± 8.9	54.2 ± 5.2	.016	Large
Percentage body fat (%)	5.9 ± 1.7	14.8 ± 2.1	< .001	Near perfect
Training data				
Weekly volume (km week^−1^)	126 ± 61	61 ± 38	.008	Large
Other training (h week^−1^)*	3.1 ± 4.9	5.8 ± 4.2	.021	Small
Training history (years)	8.3 ± 4.4	4.2 ± 2.5	.019	Medium
Performance data				
PTRS (km h^−1^)	20.1 ± 1.0	17.1 ± 0.7	< .001	Very large
Speed at VT-1 (km h^−1^)	15.6 ± 0.9	12.8 ± 0.7	< .001	Very large
Speed at RCP (km h^−1^)	17.6 ± 0.8	14.8 ± 0.9	< .001	Very large
abs VO_2peak_ (ml min^−1^ kg^−1^)	4.49 ± 0.53	3.15 ± 0.43	< .001	Very large
rel VO_2peak_ (ml min^−1^ kg^−1^)	69.8 ± 5.6	58.0 ± 4.6	< .001	Very large
VO_2_·VO_2peak_^−1^ @ VT-1 (%)	78.8 ± 4.0	79.3 ± 2.7	n.s.	–
VO_2_·VO_2peak_^−1^ @ RCP (%)	89.5 ± 3.0	90.4 ± 2.9	n.s.	–
CTT time (h:min:s)	1:14:39 ± 0:04:31	1:31:43 ± 0:06:54	< .001	Very large

* = the assumption of equality of variances was violated and thus non-parametric Mann-Whitney *U* test was performed. *Abbreviations: PTRS* peak treadmill running speed, *abs* absolute; rel = relative, *VO*_*2peak*_ peak oxygen consumption, *VT-1* first ventilatory threshold, *RCP* respiratory compensation point, *CTT* control time trial; data represented as mean ± SD

Despite the large to very large differences in anthropometric, training, and performance parameters between the sexes, there were neither significant differences in perceived fatigability and observed pacing behaviour during time trials nor in running performance after controlling for sex-dependent differences in body composition. Thus, psychophysiological responses to running in a tapered condition versus running with LMMF and EIMD are hereafter presented for female and male participants combined.

### Psychophysiological Responses to Intervention and Running Economy Test

A summary of perceptual responses to the drop-jump protocol and its subsequent impact on the isokinetic power output of knee extensors, psychophysiological responses during the running economy test, and time trial performance is provided in Table 2.

**Table 2:** Responses to drop-jump protocol and impact on power output, running economy, and time trial performance

	Control (*n* = 22)	Intervention (*n* = 22)	*p* value	Effect size
Drop-jump protocol				
Perceptual responses				
Muscle discomfort pre*	1.0 ± 0.9	0.7 ± 0.6	n.s.	–
Muscle discomfort post*	0.8 ± 0.8	2.4 ± 1.7	< 0.001	Large
Unpleasantness pre*	0.8 ± 0.7	0.8 ± 0.8	n.s.	–
Unpleasantness post*	0.8 ± 0.7	2.3 ± 1.6	< 0.001	Large
Isokinetic power output test				
Knee extension pre* (W)	152 ± 40	150 ± 45	n.s.	–
Knee extension post* (W)	150 ± 40	133 ± 45	< 0.001	Large
Running economy test				
Oxygen cost (ml kg^−1^ km^−1^)	199 ± 11	205 ± 14	0.005	Small
Energy cost (kcal kg^−1^ km^−1^)	0.99 ± 0.05	1.03 ± 0.07	0.002	Medium
Carbohydrate usage (g min^−1^)	2.74 ± 0.78	3.17 ± 0.96	0.013	Small
Heart rate (bpm)	158 ± 11	164 ± 11	0.001	Small
Perceptual responses				
Perceived physical strain	12.3 ± 1.9	13.5 ± 1.9	0.001	Medium
Perceived mental strain	6.2 ± 1.5	7.7 ± 1.6	< 0.001	Medium
Valence	2.9 ± 1.3	1.5 ± 1.6	< 0.001	Medium
Felt activation	3.8 ± 1.0	4.0 ± 0.7	n.s.	–
Time trial times				
TT time (h:min:s)	1:23:11 ± 0:10:25	1:26:37 ± 0:11:36	< 0.001	Small
2nd-half/1st-half split	0.1 ± 1.1	1.4 ± 2.2	0.003	Medium

* = simple (main) group effects (using partial eta squared as effect size measure) of two-way repeated measures ANOVA after significant treatment * time interaction effect. TT = time trial. Data represented as mean ± SD

### Time Trial Times

Times taken to complete the CTT and ITT were 1 h:23 min:11 s ± 10 min:25 s and 1 h:26 min:37 s ± 11 min:36 s, respectively. The average time difference was 3.96%. The mechanistic inference for this difference is most likely substantially negative (99.9% most likely substantially negative, 0.1% most unlikely trivial, and 0.0% most unlikely substantially positive).

### Haematological Indicators of Muscle Damage and Physiological Strain

**Fig. 1:**
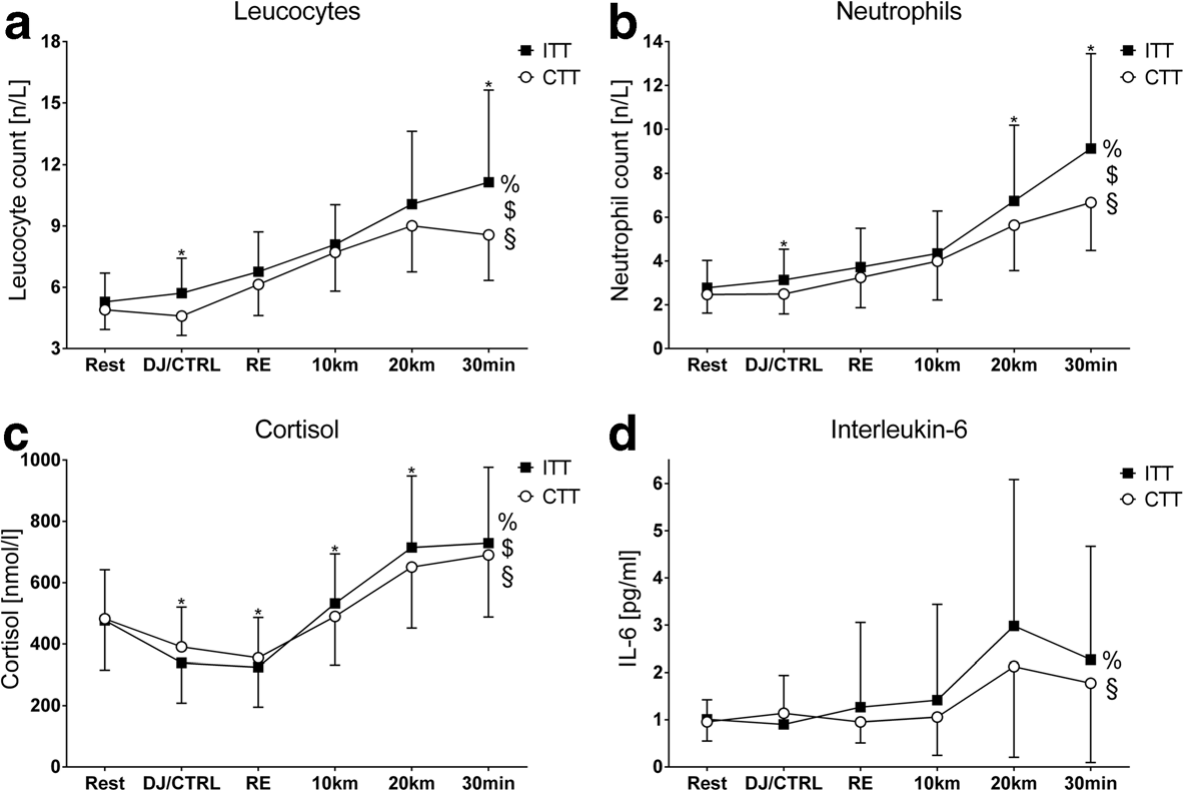
Differential responses in haematological markers of muscle damage, muscle metabolic strain, and endocrinological stress response. Panels show: **a** = blood leucocyte count; **b** = blood neutrophil count; **c** = blood cortisol concentration; **d** = blood interleukin-6 concentration. Note: blood interleukin-6 concentrations violated the assumption of normal distribution and thus non-parametric ANOVA after aligned rank transformation was conducted. Despite a significant interaction effect, simple main treatment effects did not reach significance. Abbreviations: # = main time effect; & = main group effect; % = time × group interaction effect; $ = simple (main) time effect for intervention trials; § = simple (main) time effect for control trials; * = simple (main) trial effect; ITT = intervention time trial; CTT = control time trial; DJ = drop-jump protocol; CTRL = control; RE = running economy

Graphical depictions of haematological markers of muscle damage, muscle metabolic strain, and endocrinological stress during experimental trials are summarised in Fig. 1.

### Sensory, Affective, and Cognitive Indicators of Perceived Fatigability

**Fig. 2:**
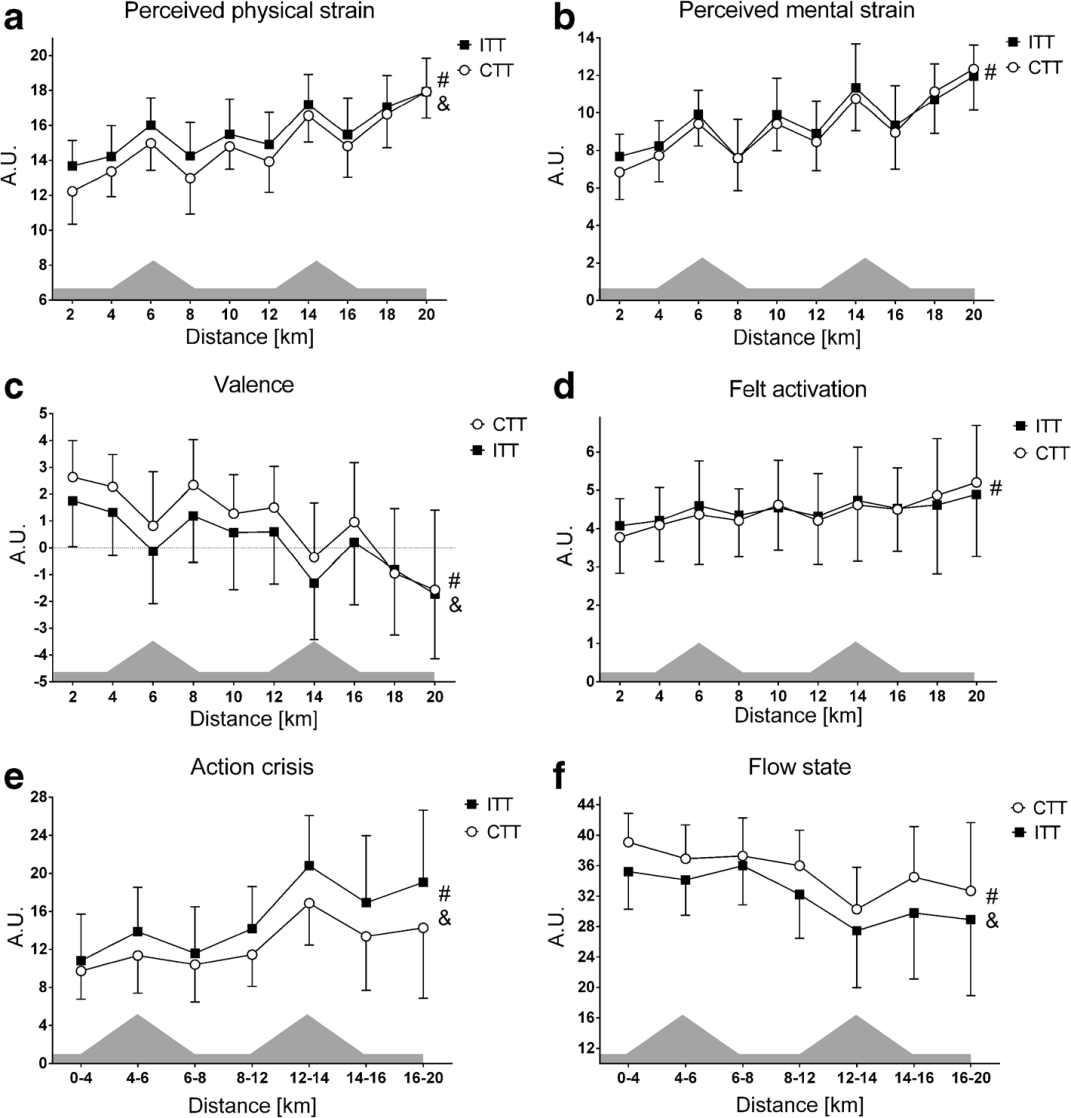
Differential responses in sensory, affective, and cognitive markers of perceived fatigability. Panels show: **a** = perceived physical strain; **b** = perceived mental strain; **c** = valence; **d** = felt activation; **e** = action crisis; **f** = flow state. Note the differences in the *x*-axes of action crisis and flow state due to different sampling times. The shaded topography represents the course profile of the 20-km treadmill time trial. Abbreviations: # = main time effect; & = main trial effect; ITT = intervention time trial; CTT = control time trial

Graphical depictions of differential responses in sensory-discriminatory, affective-motivational, and cognitive-evaluative indicators of perceived fatigability during experimental trials are summarised in Fig. 2.

### Indicators of Performance Fatigability

**Fig. 3:**
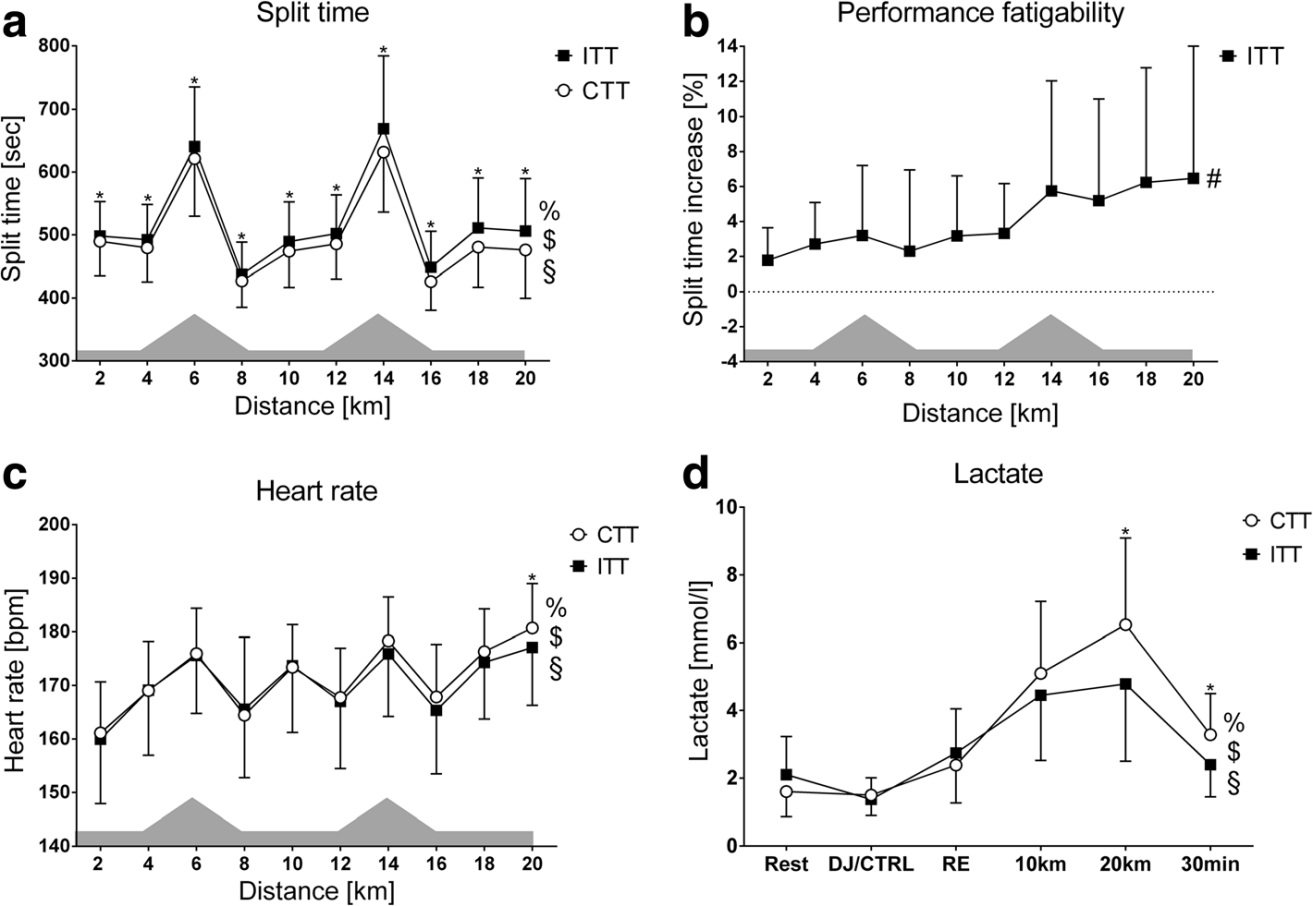
Differential responses in markers of performance fatigability. Panels show: **a** = split time; **b** = performance fatigability; **c** = heart rate; **d** = blood lactate concentration. Note the differences in the *x*-axes of blood lactate concentrations due to different sampling times. Performance fatigability is indicated by the percentage increase in split time during the intervention time trial compared to control time trial and assessed via one-way repeated measures ANOVA. The shaded topography represents the course profile of the 20-km treadmill time trial. Abbreviations: # = main time effect; % = time × trial interaction effect; $ = simple (main) time effect for intervention trials; § = simple (main) time effect for control trials; * = simple (main) trial effect; ITT = intervention time trial; CTT = control time trial; DJ = drop-jump protocol; CTRL = control; RE = running economy

Graphical depictions of differential responses in markers of performance fatigability are summarised in Fig. 3.

### Performance, Perceptive, and Haematological Data

A summary of statistical findings in main performance, perceptive, and haematological variables is provided in Table 3.

**Table 3:**
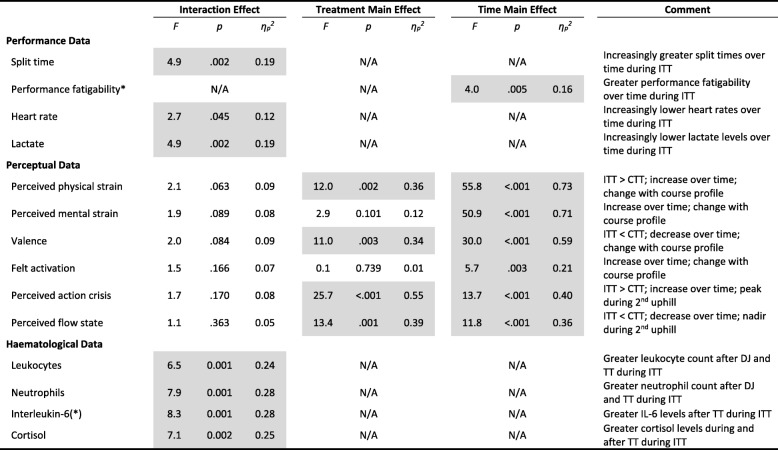
Summary of treatment, time, and interaction effects in main performance, perceptive, and haematological variables

* = performance fatigability is indicated by the relative percentage increase in split times during ITT compared to CTT and assessed via one-way repeated measures ANOVA. (*) = blood interleukin-6 concentrations violated the assumption of normal distribution and thus non-parametric ANOVA after aligned rank transformation was conducted. For details, see Additional file 1. Note: shaded areas significant at *p* < .05; TT = time trial; ITT = intervention time trial; CTT = control time trial; DJ = drop-jump protocol

Due to space restrictions, the following results are provided in Additional file 1 only. Findings of deterioration in jump-height during the drop-jump protocol and the development of DOMS in the 84-h recovery period after experimental trials are provided in Additional file 1: SM1. The distributions of individual differences in knee extensor power output, time trial time, and time trial splits are provided in Additional file 1: SM2. Statistical details of two-way repeated measures ANOVA’s (trial × time) including follow-up tests and non-parametric ANOVA’s in perceptive variables are provided in Additional file 1: SM3 and results of three-way mixed repeated measures ANOVA’s (trial × time × sex) are provided in Additional file 1: SM4.

## Discussion

According to Enoka and Duchateau [24], human fatigue is a psychophysiological symptom underpinned by interactions between (A) performance fatigability—the observed decline in an objective measure of endurance performance over a discrete period of time—and (B) perceived fatigability—changes in the perceptions that regulate the integrity of the performer based on the maintenance of homeostasis and the psychological state of the individual. The main findings of the current study indeed provide supportive evidence for physiological and perceptual mechanisms underlying observed pacing behaviour and performance fatigability.

### Physiological Effects of LMMF and Mild EIMD on Performance Fatigability

First, the muscle-lengthening contraction protocol induced the desired muscle damage and reduction in power output generating the capacity of locomotor muscles (see Additional file 1: SM1) without confounding effects of significant muscle metabolite accumulation and cardiovascular stress. The large decrease of 11% in isokinetic strength is consistent with mild EIMD [10] (see Table 2), but only about half the decrease in isometric strength was observed in other studies [13, 29]. Although not directly comparable, the lesser strength loss is best explained by the high-performance calibre of our participants and the ‘repeated bout effect’ rendering runners more resistant to muscle damage induced by muscle-lengthening contractions than are moderately trained cyclists.

Second, running with LMMF and mild EIMD stimulated amplified responses in cardiovascular, respiratory, and metabolic variables during the running economy test. A small increase in heart rate, oxygen consumption, and carbohydrate usage (including concomitant trend towards decreased fat usage) as well as a medium increase in energy cost of running collectively point towards increased physiological demands (see Table 2). Thus, running with LMMF and mild EIMD at ∆ 1/3 between the first ventilatory threshold and respiratory compensation point (i.e. close to long-distance ‘race-pace’) increased absolute physiological strain and relative exercise intensity. Potential causes of augmented responses are manifold and, for example, could stem from inefficient alterations to running kinematics [18, 21, 22], increased afferent feedback from groups III and IV muscle afferent fibres resulting from structural disruption in the extrafusal muscle fibres and local microvasculature [52, 53], and increased compensatory efferent motor command to weakened locomotor muscles [13, 29]. Both, afferent feedback and efferent feedforward components therefore seem to impact and modulate physiological responses and collectively support the notion of an underlying reafference principle in the allostatic regulation of homeostasis [30].

Third, medium increases in blood leucocyte and neutrophil counts, interleukin-6, and cortisol concentrations were found during the ITT, respectively, indicating greater mobilisation and migration to damaged tissue (and thus indirectly EIMD), greater muscle metabolic strain [54, 55], and endocrinological stress (see Fig. 1 and Table 3). The absolute values in these variables closely resemble those observed after ‘real-world’ half marathon events [56]. Together, leucocytosis, neutrophilia, and pro-inflammatory immune response reflect the exacerbated physiological strain of running with LMMF and mild EIMD [57, 58]. Additionally, endocrinological stress response during the ITT was aggravated despite substantial performance decrement, thereby clearly indicating a non-adaptive distress response [59, 60]. Collectively, the previously described responses constitute a physiological milieu that is likely not conducive to high performance as evidenced by the one-tailed detrimental effects of LMMF and mild EIMD on exercise performance (see Additional file 1: SM2).

Accordingly, we suggest that muscle damage and the amplified physiological responses to running with LMMF and mild EIMD per se have a debilitating effect on endurance performance due to increased absolute physiological strain and relative exercise intensity conveyed by peripheral and central fatigue mechanisms [61, 62]. Time trial performance during ITT was constrained from the start indicating restricted work capacity and, potentially underpinned by accumulating EIMD, deteriorated further excessively from the second uphill onwards (see Fig. 3). Del Coso et al. [63] observed a similar differential pattern of pacing behaviour in marathon runners either maintaining (i.e. even split) or reducing (i.e. positive split) running speed and found significant correlations between increased performance fatigability and indirect haematological markers of muscle damage. However, running with LMMF and mild EIMD also elicited potentially negative effects on performance fatigability via deterioration in sensory, affective, and cognitive processes hypothesised to underpin perceived fatigability.

### Perceptual Effects of LMMF and Mild EIMD on Performance Fatigability

First, a large increase in perceived muscle discomfort was found in response to the muscle-lengthening contractions protocol, although absolute scores are still relatively mild. In addition, medium increases in perceived physical and mental strain were observed in response to running with LMMF and mild EIMD during the running economy test (see Table 1). Perceived physical and mental strain showed a trend towards greater perceived strain scores during the start of the ITT, but eventually approached similar end-points (see Fig. 2). This is coherent with the findings from de Morree et al. [29] suggesting that both are primary regulatory variables of trajectory pacing behaviour [44].

A large strain construct × treatment interaction effect (*F*_1,21_ = 10.1; *p* = .005; ${\eta}_p^2$ = 0.32) was observed with a large main treatment effect for greater perceived physical strain only during the ITT (see Table 3). This points towards the accuracy of perceived physical strain in the psychophysiological integration of homeostatic disturbance and the ability of athletes to differentiate between perceived physical strain and perceived mental strain [64, 65]. Further empirical support was recently provided by Girard et al. [66], who observed associations between increased physiological disturbance and subsequent augmented exercise-related physical sensations and performance fatigability during mental effort-clamped ‘all-out’ sprints in hypoxia compared to normoxia. This suggests that under conditions of intensified muscular, respiratory, and/or thermal strain, perceived physical strain can dissociate from perceived mental strain and may play a mediatory role in performance regulation.

Second, a large decrease in valence in response to the muscle-lengthening protocol and a medium decrease in response to running with LMMF and mild EIMD during the running economy test (see Table 2) was associated with increases in perceived strain variables. Importantly, a large main treatment effect of attenuated valence during the ITT was associated with a large main treatment effect of augmented perceived physical strain (see Fig. 2). This attachment of valence to interoceptive stimuli facilitates the awareness of homeostatic disturbance and confirms the paramount importance of valence in interoception, awareness, and computation of conflicting motivational drives [67, 68]. Thus, valence is suggested to be the driving force by which attentional focus is shifted from goal-driven towards stimulus-driven processes. It thereby functions as an attractor in attention allocation and decision-making, and ultimately performance regulation [69, 70].

Third, the decrease of valence during the ITT was associated with large increases and decreases in action crisis and flow state, respectively (see Fig. 2). An action crisis has been defined as an intra-psychic conflict between further goal pursuit and goal disengagement resulting from negatively valenced events in goal striving [71]. In an action crisis, athletes deliberate once again the desirability and feasibility of the pursued and alternative goals, thereby undermining effective goal striving [72]. In contrast, flow has been described as a state of optimal experience, where athletes are totally immersed in goal striving and goal attainment [73]. Accordingly, their mirror-like dynamic responses suggest they form opposing ends of the mindset spectrum [74] and clearly identify a shift from an implemental mindset cognitively tuned towards the ‘how’ of behaviour to a deliberative mindset cognitively tuned towards the ‘why’ of behaviour.

Critically, increased cost-benefit thinking of further goal pursuit and goal disengagement has been shown to vary independently from the linearly increasing physiological strain experienced during a marathon, thereby independently predicting performance decrement [72, 75]. Within the sport domain, mediation analyses showed that an action crisis undermines performance by augmenting the endocrinological distress response [31, 72], while cross-lagged panel path analyses in the academic domain showed attenuation of goal desirability and perceived goal attainability [72, 76]. Thus, an action crisis can be understood as an antecedent in the goal disengagement process by draining physiological and self-regulatory resources, eventually leading to the dissolution of the pursued goal.

In support, during the second climb of the ITT, the large deterioration and peak in action crisis indeed coincided with an excessive increment in performance fatigability, thereby suggesting that runners were experiencing a decisional conflict between further goal pursuit and goal disengagement just before they (had to) made the decision to slow-down.^3^ Supportive evidence (although only associative) for the notion that runners re-evaluated and partly disengaged from their focal goal comes from the observations of increasingly lower heart rates and blood lactate concentrations towards the finish (see Fig. 3) as well as perceptual inflexions towards more sustainable rates of change in valence and action crisis after the second climb (see Fig. 2).

## Conclusions

The current findings demonstrate that running with LMMF and mild EIMD caused medium-sized physiological and large-sized perceptual effects that were associated with discrete and poignant onset of unintended alteration in performance fatigability and observed pacing behaviour, thereby closely resembling defining characteristics of the ‘hitting the wall’ phenomenon [77, 78]. It is further suggested that much of the variance in response to running with LMMF and mild EIMD can be explained by the dynamic and complex interactions between the investigated psychophysiological determinants of pacing behaviour and performance during prolonged endurance exercise. Both physiological and perceptual pathways are proposed to impact performance fatigability during running with LMMF and mild EIMD via (1) amplified physiological strain and non-adaptive endocrinological distress response and (2) increase in perceived fatigability. Specifically with regards to the latter, it is hypothesised that an increase in perceived physical strain *antecedes* a decrease in valence, which in turn *antecedes* an increase in action crisis, eventually dissolving the initially aspired goal. The applied three-dimensional framework of perceived fatigability therefore provides a more comprehensive understaning of strain-perception-thinking-action coupling in centrally regulated and goal-directed exercise behvaiour than the traditional Gestalt concept of perceived exertion [79].

The extent to which the current data fit, the hypothesised cause-effect relationships has been assessed separately in a subsequent companion article using a five-step structural equation modelling procedure [80]. 

## Additional File

Additional file 1:  Supplementary material containing findings in (1) deterioration in jump-height during the drop-jump protocol and the development of DOMS in the 84-h recovery period after experimental trials, (2) distributions of individual differences in knee extensor power output, time trial time, and time trial splits, (3) statistical details of two-way repeated measures ANOVA’s (trial × time) including follow-up tests and non-parametric ANOVA’s in perceptive variables, and (4) results of three-way mixed repeated measures ANOVA’s (trial × time × sex). (PDF 317 kb)

## Abbreviations

ACRISS: Action Crisis Scale
ANOVA: Analysis of variance
BTT: Baseline time trial
COM: Centre of mass
CTT: Control time trial
DOMS: Delayed onset of muscular soreness
EDTA: Ethylenediaminetetraacetic acid
EIMD: Exercise-induced muscle damage
ELISA: Enzyme-linked immunosorbent assay
FAS: Felt Arousal Scale
FS: Feeling Scale
FSS: Flow State Scale
FTT: Familiarisation time trial
ITT: Intervention time trial
LMMF: Locomotor muscle fatigue
PTRS: Peak treadmill running speed
RCP: Respiratory compensation point
SM: Supplementary material
TOV: Take-off velocity of COM
VAS: Visual analogue scale
VCO_2_: Carbon dioxide production
V_E_: Expired ventilation volume
VO_2_: Oxygen uptake
VO_2peak_: Peak oxygen uptake
VT-1: First ventilatory threshold

## Footnotes

1 Reafference is the anticipated sensory stimulation elicited by voluntary movements and is linked to efferent motor command. It thereby allows distinction between self-initiated sensory stimulation from external stimuli, so called exafference. The computation of the error difference underpins the reafference principle [81].

2 Corrected total-item correlation of item eight (transformation of time) was consistently negative throughout both trials indicating that item 8 loads negatively on the overall flow state construct. Removal of item 8 would have been justified and significantly improved the internal consistency estimate of reliability on average from 0.81 ± 0.06 to 0.91 ± 0.04. However, given the small sample size and the nevertheless good Cronbach’s alpha, item 8 was kept, but it is recommended that critical revision of item 8 of the flow state scale is required. For a recent critical review on flow in sports and exercise, see Swann et al. [82].

3 A strength of this study was the use of a custom-made handheld remote control in the investigation of maximal self-paced treadmill running behaviour. In contrast to cycling ergometer studies, where it is unclear whether participants intentionally or unintentionally change pace, runners in this study decisively had to use the remote control to adjust treadmill running speed. However, it remains unclear whether this decision was based on heuristic or rational computations, or a combination thereof [83]. The “(had to)” refers to the hypothesised driving role of valence in an affect heuristic-triggered decision-making process.
