# Supplementary material for: The Psychophysiological Regulation of Pacing Behaviour and Performance Fatigability During Long-Distance Running with Locomotor Muscle Fatigue and Exercise-Induced Muscle Damage in Highly Trained Runners
Source: Sports Med Open. 2018 Jul 10;4:29. doi: 10.1186/s40798-018-0143-2 (PMC6037655; doi:10.1186/s40798-018-0143-2)
Supplement: Supplementary file 1 — Supplementary material containing findings in (1) deterioration in jump-height during the drop-jump protocol and the development of DOMS in the 84-h recovery period after experimental trials, (2) distributions of individual differences in knee extensor power output, time trial time, and time trial splits, (3) statistical details of two-way repeated measures ANOVA’s (trial × time) including follow-up tests and non-parametric ANOVA’s in perceptive variables, and (4) results of three-way mixed repeated measures ANOVA’s (trial × time × sex). (PDF 317 kb) [file 40798_2018_143_MOESM1_ESM.pdf]

SUPPLEMENTARY MATERIAL

Title

The psychophysiological regulation of pacing behaviour and performance fatigability during long-distance running with locomotor muscle fatigue and exercise-induced muscle damage in highly-trained runners

Journal

Sports Medicine - Open

Corresponding author:

Andreas Venhorst
Department of Human Biology, University of Cape Town, Division of Exercise Science & Sports Medicine, Newlands 7725, South Africa;
andreas.venhorst@gmail.com

Co-author(s):

Dominic P. Micklewright
School of Sport, Rehabilitation and Exercise Sciences, University of Essex, Colchester UK. CO4 3SQ

Timothy D. Noakes
Department of Human Biology, University of Cape Town, Division of Exercise Science & Sports Medicine, Newlands 7725, South Africa;

SUPPLEMENTARY MATERIAL ONE

Drop-Jump Protocol

Jump-Height

There was a highly significant effect of time on jump-height during the drop-jump protocol ($F_{3.5, 72.5}$=9.2; p=0.000; η_p^2=0.31) with significantly lower jump-heights after eighty drop-jumps.

Delayed Onset of Muscular Soreness (DOMS)

There was a significant treatment × time interaction effect on DOMS ($F_{2.6, 55.4}$=4.4; p=0.010; η_p^2=0.17). The investigation of simple (main) treatment effects showed highly significantly greater DOMS at every 12-hour interval ($F_{1, 21}$>18.5; p<0.001; η_p^2 >0.47) peaking 36-hours after the intervention ($F_{1, 21}$=198.1; p<0.001; η_p^2>0.90). DOMS changed highly significantly during the recovery from the intervention ($F_{3.4, 71.5}$=20.3; p=0.000; η_p^2=0.49) as well as the control time trial ($F_{2.8, 58.6}$=28.6; p=0.000; η_p^2=0.58).

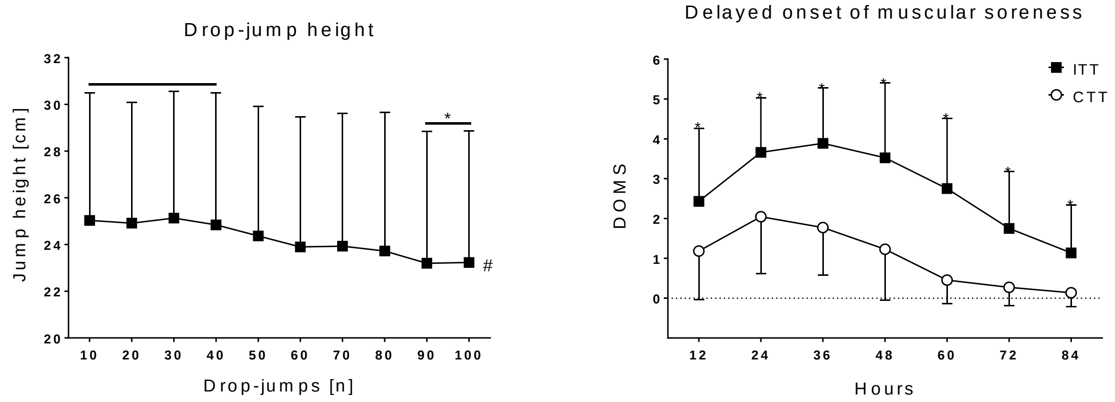

Figure SM1 Decline in jump height over time during the drop-jump protocol and development of delayed onset of muscular soreness

SUPPLEMENTARY MATERIAL TWO

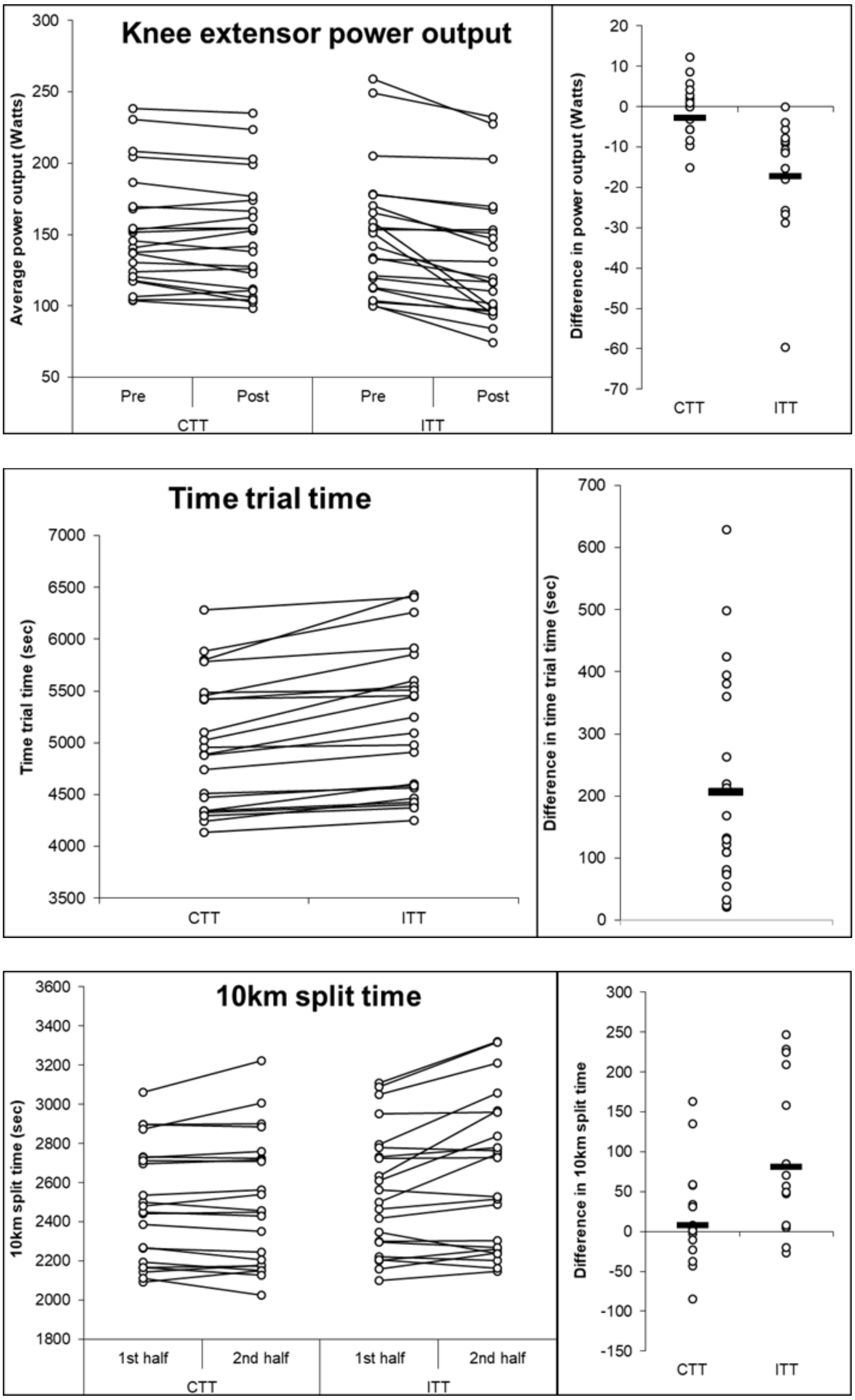

Figure SM2 Distribution of individual responses to drop-jump protocol in knee extensor power output, 20 km time trial time, and 10 km time trial splits

SUPPLEMENTARY MATERIAL THREE

Detailed Results of Two-Way (WW) RM ANOVA in Main Outcome Variables of Interest

Performance Data

Split Time

There was a highly significant treatment × time interaction effect for 2 km split times ($F_{3.6, 76.4}$=4.9; p=0.002; η_p^2=0.19). The investigation of simple (main) treatment effects showed that runners took significantly more time during each 2-km section of the intervention trial. Except for the first downhill section ($F_{1, 21}$=5.2; p=0.033; η_p^2=0.20), all 2 km split times were highly significantly greater compared to the control trial ($F_{1, 21}$>13.9; p<0.001; η_p^2>0.40). Split times changed highly significantly in accordance with course profile in both treatment groups with simple (main) time effects of ($F_{1.7, 35.7}$=229.6; p=0.000; η_p^2=0.92) in the control ($F_{2.1, 44.1}$=161.4; p=0.000; η_p^2 =0.89) and intervention trial, respectively.

Performance Fatigability

There was a highly significant main time effect for performance fatigability expressed as percentage increase in split time during the intervention time trial compared to the control time trial and was assessed by means of one-way repeated measures analysis of variance ($F_{4.1, 83.5}$=4.0; p=0.005; η_p^2=0.16). The investigation of simple (main) time effects found no significant effects at 2-km intervals.

Heart Rate

There was a significant treatment × time interaction effect for average heart rate at 2-km intervals ($F_{3.3, 68.8}$=2.7; p=0.045; η_p^2=0.12). The investigation of simple (main) treatment effects showed a trend for significantly lower heart rates in runners during the intervention trial from the second climb onwards ($F_{1, 21}$=3.8; p=0.066; η_p^2=0.15) and this reached highly statistical significance during the final 2-km segment compared to the control trial ($F_{1, 21}$=9.5; p=0.006; η_p^2=0.31). Average heart rates increased highly significantly over time and changed according to course profile in both treatment

groups with simple (main) time effects of ($F_{3.8, 80.3}$=71.4; p=0.000; η_p^2=0.77) in the control ($F_{3.5, 61.9}$=53.0; p=0.000; η_p^2=0.72) and intervention trial, respectively.

Perceptive Data

All perceptive data were additionally analysed with a non-parametric factorial procedure (ART) using common analysis of variance after align rank transform. The results are provided in squared brackets after the parametric test results in parenthesis.

Perceived Physical Strain (6-20 Borg scale)

There was a trend for a treatment × time interaction effect in perceived physical strain during the intervention trial with higher scores during the start phase and increasingly similar scores towards the end ($F_{5.4, 113.0}$=2.1; p=0.063; η_p^2=0.09) [ART: $F_{6.0, 126.8}$=1.8; p=0.104; η_p^2=0.08]. However, the main treatment effect was highly significant with greater perceived physical strain during the intervention trial compared to the control trial ($F_{1, 21}$=12.0; p=0.002; η_p^2=0.36) [ART: $F_{1, 21}$=9.9; p=0.005; η_p^2=0.32]. Perceived physical strain increased highly significantly over time and in accordance with course profile in both control and intervention trial ($F_{3.6, 75.5}$=55.8; p=0.000; η_p^2=0.73) [ART: $F_{3.9, 81.1}$=53.8; p=0.000; η_p^2=0.72].

Perceived Mental Strain (0-14 Borg scale)

There was a trend for a treatment × time interaction effect in perceived mental strain during the intervention trial with higher scores during the start phase and increasingly similar scores towards the end ($F_{5.5, 115.4}$=1.9; p=0.089; η_p^2=0.08) [ART: $F_{6.1, 127.8}$=2.0; p=0.064; η_p^2=0.09]. Neither was there a significant main treatment effect between the intervention and control trial ($F_{1, 21}$=2.9; p=0.101; η_p^2=0.12) [ART: $F_{1, 21}$=2.2; p=0.154; η_p^2=0.09]. However, perceived mental strain increased highly significantly over time and in accordance with course profile in both control and intervention trial ($F_{3.7, 78.6}$=50.9; p=0.000; η_p^2=0.71) [ART: $F_{4.5, 94.4}$=54.9; p=0.000; η_p^2=0.72].

Feeling Scale (FS)

There was a trend for a treatment × time interaction effect in valence during the intervention trial with lower scores during the start phase and increasingly similar scores towards the end ($F_{4.7, 99.0}$=2.0; p=0.084; η_p^2=0.09) [ART: $F_{4.6, 96.6}$=1.9; p=0.111; η_p^2=0.08]. However, the main treatment effect was highly significant with greater negative valence during the intervention trial compared to the control trial ($F_{1, 21}$=11.0; p=0.003; η_p^2=0.34) [ART: $F_{1, 21}$=8.5; p=0.008; η_p^2=0.26]. Valence deteriorated highly significantly over time and changed in accordance with course profile in both control and intervention trial ($F_{3.0, 63.7}$=30.0; p=0.000; η_p^2=0.59) [ART: $F_{3.6, 75.1}$=28.9; p=0.000; η_p^2=0.58].

Felt Arousal Scale (FAS)

There was neither a significant treatment × time interaction effect ($F_{9, 189}$=1.5; p=0.166; η_p^2=0.07) [ART: $F_{5.5, 115.0}$=1.4; p=0.231; η_p^2=0.06], nor a main treatment effect for felt activation ($F_{1, 21}$=0.1; p=0.739; η_p^2=0.01) [ART: $F_{1, 21}$=0.3; p=0.574; η_p^2=0.01]. However, felt activation increased highly significantly over time and according to course profile in both control and intervention trial ($F_{2.6, 54.5}$=5.7; p=0.003; η_p^2=0.21) [ART: $F_{2.9, 75.1}$=11.8; p=0.000; η_p^2=0.36].

Action Crisis Scale (ACRISS)

There was no significant treatment × time interaction effect for perceived action crisis ($F_{3.2, 66.5}$=1.7; p=0.170; η_p^2=0.08) [ART: $F_{3.3, 69.3}$=2.3; p=0.075; η_p^2=0.10]. However, the main treatment effect was highly significant with greater perceived action crisis during the intervention trial compared to the control trial ($F_{1, 21}$=25.7; p=0.000; η_p^2 =0.55) [ART: $F_{1, 21}$=27.6; p=0.000; η_p^2=0.57]. Perceived action crisis increased highly significantly over time and in accordance with course profile reaching peak values during the second climb in both control and intervention trial ($F_{2.2, 45.5}$=13.7; p=0.000; η_p^2=0.40) [ART: $F_{2.3, 47.7}$=13.3; p=0.000; η_p^2=0.39].

Flow State Scale – short (FSS)

There was no significant treatment × time interaction effect for perceived flow state ($F_{3.2,\ 66.6}$=1.1; p=0.363; η_p^2=0.05) [ART: $F_{3.6,\ 75.5}$=0.9; p=0.475; η_p^2=0.04]. However, the main treatment effect was highly significant with reduced perceived flow state during the intervention trial compared to the control trial ($F_{1,\ 21}$=13.4; p=0.001; η_p^2 =0.39) [ART: $F_{1,\ 21}$=12.9; p=0.002; η_p^2=0.38]. Perceived flow state decreased highly significantly over time reaching minimum values during the second climb in both control and intervention trial ($F_{2.1,\ 44.5}$=11.8; p=0.000; η_p^2=0.36) [ART: $F_{2.3,\ 46.8}$=10.4; p=0.000; η_p^2=0.33].

Haematological Data

Lactate

There was a highly significant treatment × time interaction effect for blood lactate concentrations ($F_{3.6,\ 76.4}$=4.9; p=0.002; η_p^2=0.19). The investigation of simple (main) treatment effects showed that runners had highly significantly lower blood lactate concentrations at the finish of ($F_{1,\ 21}$=9.4, p=0.006; η_p^2=0.31) and after 30 minutes of recovery from ($F_{1,\ 21}$=11.7, p=0.003; η_p^2=0.36) the 20-km intervention time trial compared to the control time trial. Blood lactate concentrations increased highly significantly over time peaking at the finish of the 20-km time trial in both treatment groups with simple (main) time effects of ($F_{2.3,\ 48.5}$=53.1; p=0.000; η_p^2=0.72) in the control trial and ($F_{2.9,\ 60.7}$=30.4; p=0.000; η_p^2=0.59) in the intervention trial, respectively.

Cortisol

There was a highly significant treatment × time interaction effect for blood cortisol concentrations ($F_{2.1,\ 43.8}$=7.1; p=0.002; η_p^2=0.25). The investigation of simple (main) treatment effects showed that runners had significantly lower blood cortisol concentrations after the drop-jump protocol ($F_{1,\ 21}$=12.5; p=0.002; η_p^2=0.37) and the running economy assessment ($F_{1,\ 21}$=4.7; p=0.042; η_p^2=0.18), but significantly greater blood cortisol concentrations after 10 km ($F_{1,\ 21}$=4.4; p=0.048; η_p^2=0.17) and at the

finish of the 20-km intervention time trial ($F_{1, 21}$=7.0; p=0.016; η_p^2=0.25) compared to the control time trial. Blood lactate concentrations increased highly significantly over time peaking after 30 min of recovery in both treatment groups with simple (main) time effects of ($F_{2.3, 49.1}$=89.0; p=0.000; η_p^2=0.81) in the control trial and ($F_{2.0, 42.3}$=111.2; p=0.000; η_p^2=0.84) in the intervention trial, respectively.

Interleukin-6

The assumption of normal distribution in blood interleukin-6 concentrations was violated. Thus, non-parametric analysis of variance after aligned rank transformation was conducted. There was a highly significant treatment × time interaction effect for blood interleukin-6 concentrations (ART: $F_{2.1, 43.6}$=8.3; p=0.001; η_p^2=0.28). However, simple (main) treatment effects did not reach statistical significance. Blood interleukin-6 concentrations increased significantly over time and peaked at the finish of the 20-km time trials respectively increasing by two-fold ($F_{1.8, 38.2}$=5.2; p=0.012; η_p^2=0.20) and three-fold ($F_{3.0, 62.4}$=25.0; p=0.000; η_p^2=0.54) during the control and intervention trial.

Leucocytes

There was a highly significant treatment × time interaction effect for blood leucocyte count ($F_{2.6, 55.2}$=6.5; p=0.001; η_p^2=0.24). The investigation of simple (main) treatment effects showed that runners had highly significantly greater blood leucocyte count after the drop-jump protocol ($F_{1, 21}$=16.2; p=0.001; η_p^2=0.44) and after 30 minutes of recovery (F1, 21=14.5; p=0.001; η_p^2=0.41) from the intervention time trial compared to the control time trial. Blood leucocyte concentrations increased highly significantly over time peaking after 30 min of recovery in both treatment groups with simple (main) time effects of ($F_{2.4, 50.0}$=53.4; p=0.000; η_p^2=0.72) in the control trial and ($F_{1.7, 35.1}$=37.9; p=0.000; η_p^2=0.64) in the intervention trial, respectively.

Neutrophils

There was a highly significant treatment × time interaction effect for blood neutrophil count ($F_{2.0, 43.0}$=7.9; p=0.001; η_p^2=0.28). The investigation of simple (main) treatment

effects showed that runners had significantly greater blood neutrophil count after the drop-jump protocol ($F_{1, 21}$=12.1; p=0.002; η_p^2=0.37), at the finish of the 20-km time trial ($F_{1, 21}$=4.5; p=0.045; η_p^2=0.18) and after 30 minutes of recovery (F1, 21=16.16; p=0.001; η_p^2=0.44) from the intervention time trial compared to the control time trial. Blood neutrophil count increased highly significantly over time peaking after 30 min of recovery in both treatment groups with simple (main) time effects of ($F_{2.3, 49.2}$=57.3; p=0.000; η_p^2=0.73) in the control trial and ($F_{1.6, 31.6}$=46.9; p=0.000; η_p^2=0.69) in the intervention trial, respectively.

SUPPLEMENTARY MATERIAL FOUR

Detailed Results of Three-Way (WWB) RM ANOVA in Main Outcome Variables of Interest

Performance Data

There were no significant treatment × time × sex interaction effects for split time ($F_{3.6, 72.3}$=0.8; p=0.531; η_p^2=0.04), performance fatigability ($F_{3.8, 76.2}$=0.9; p=0.479; η_p^2 =0.04) or heart rate ($F_{3.1, 61.9}$=0.8; p=0.490; η_p^2=0.04).

However, significant main sex effects were observed for split time ($F_{1, 20}$=51.6; p=0.000; η_p^2=0.72). These effects disappeared after controlling for sex dependent differences in body fat percentage ($F_{1, 19}$=0.6; p=0.432; η_p^2=0.03).

Perceptive Data

All perceptive data were additionally analysed with a non-parametric factorial procedure (ART) using common analysis of variance after align rank transform. The results are provided in squared brackets after the parametric test results in parenthesis.

There were no significant treatment × time × sex interaction effects for perceived physical strain ($F_{5.3, 105.1}$=1.4; p=0.248; η_p^2=0.06) [ART: $F_{9, 180}$=1.4; p=0.206; η_p^2 =0.06], perceived mental strain ($F_{5.4, 108.3}$=0.9; p=0.475; η_p^2=0.04) [$F_{9, 180}$=1.3; p=0.233; η_p^2=0.06], valence ($F_{4.5, 90.8}$=0.8; p=0.570; η_p^2=0.04) [ART: $F_{4.5, 89.5}$=0.9; p=0.487; η_p^2=0.04], felt activation ($F_{9, 180}$=0.8; p=0.585; η_p^2=0.04) [ART: $F_{5.4, 108.5}$=0.5; p=0.761; η_p^2=0.03], perceived action crisis ($F_{3.3, 65.5}$=1.9; p=0.125; η_p^2=0.09) [ART: $F_{3.5, 69.2}$=1.7; p=0.160; η_p^2=0.08] and perceived flow state ($F_{3.2, 64.6}$=1.1; p=0.378; η_p^2 =0.05) [ART: $F_{3.6, 71.4}$=1.1; p=0.402; η_p^2=0.05].

There were also no main sex effects for perceived physical strain ($F_{1, 20}$=0.3; p=0.591; η_p^2=0.02) [ART: $F_{1, 20}$=0.2; p=0.625; η_p^2=0.01], perceived mental strain ($F_{1, 20}$=0.9; p=0.349; η_p^2=0.04) [ART: $F_{1, 20}$=0.8; p=0.383; η_p^2=0.04], valence ($F_{1, 20}$=0.6; p=0.447;

η_p^2=0.03) [ART: $F_{1, 20}$=0.5; p=0.486; η_p^2=0.03], felt activation ($F_{1, 20}$<0.0; p=0.961; η_p^2 <0.00) [ART: $F_{1, 20}$<0.0; p=0.979; η_p^2<0.00], perceived action crisis ($F_{1, 20}$<0.0; p=0.917; η_p^2<0.00) [ART: $F_{1, 20}$<0.0; p=0.845; η_p^2<0.00] or perceived flow state ($F_{1, 20}$=0.6; p=0.430; η_p^2=0.03) [ART: $F_{1, 20}$=0.1; p=0.728; η_p^2=0.01].

Haematological Data

There were no significant treatment × time × sex interaction effects for blood concentrations in lactate ($F_{2.8, 55.9}$=0.4; p=0.771; η_p^2=0.02), cortisol ($F_{2.0, 39.9}$=1.2; p=0.306; η_p^2=0.06) and interleukin-6 (ART: $F_{2.0, 40.3}$=1.6; p=0.218; η_p^2=0.07), but there were trends towards greater blood cell count for female runners during the intervention trial in leucocytes ($F_{2.9, 57.3}$=2.5; p=0.067; η_p^2=0.11) and neutrophils ($F_{2.2, 44.0}$=2.6; p=0.077; η_p^2=0.11).

However, significant main sex effects with greater blood concentration in female runners were observed in cortisol ($F_{1, 20}$=7.5; p=0.013; η_p^2=0.27) as well as greater leucocyte ($F_{1, 20}$=4.7; p=0.042; η_p^2=0.19) neutrophil counts ($F_{1, 20}$=3.9; p=0.063; η_p^2 =0.16). These effects disappeared after controlling for sex dependent differences in weekly mileage for cortisol ($F_{1, 19}$=1.1; p=0.313; η_p^2=0.05), leucocytes (ANCOVA: $F_{1, 19}$=0.7; p=0.426; η_p^2=0.03) and neutrophils (ANCOVA: $F_{1, 19}$=0.3; p=0.589; η_p^2 =0.02), respectively.
